# Development of Cracks in the Ice Resources for Aboriginal and Torres Strait Islander Peoples, a Culturally Appropriate Evidence-Based Online Community Toolkit About Crystal Methamphetamine: Mixed Methods Study

**DOI:** 10.2196/75957

**Published:** 2026-08-21

**Authors:** Steph Kershaw, Katie Dean, Jessica Deng, Tariq Hill, Louise Birrell, Kate Ross, Lexine A Stapinski, Nicola C Newton, Katrina E Champion, Andrew Amor, Dennis Gray, Kimberly Cartwright, Steve Allsop, Nyanda McBride, Frances Kay-Lambkin, Maree Teesson, Cath Chapman, Scott Wilson

**Affiliations:** 1 The Matilda Centre for Research in Mental Health and Substance Use, The University of Sydney, Level 6 G02 Jane Foss Russell Building, Sydney, 2006, Australia, 61 (02)86279048; 2 Milliya Rumurra Aboriginal Corporation, Broome, Australia; 3 National Drug Research Institute, Faculty of Health Sciences, Curtin University, Perth, Australia; 4 Hunter Medical Research Institute, Newcastle, Australia; 5 Aboriginal Drug & Alcohol Council, Adelaide, Australia

**Keywords:** methamphetamine, co-development, substance-related disorders, health education, Aboriginal and Torres Strait Islander, mobile phone

## Abstract

**Background:**

Crystal methamphetamine (“ice”) is a drug of significant concern across Australia; however, access to culturally appropriate, community-led, and co-developed resources is limited for Aboriginal and Torres Strait Islander communities.

**Objective:**

To meet this need, the Cracks in the Ice Resources for Aboriginal and Torres Strait Islander peoples web-based toolkit was developed to support Aboriginal and Torres Strait Islander peoples, families, and communities affected by crystal methamphetamine.

**Methods:**

The community-led development of the toolkit was overseen by an expert advisory group and involved a broad-reaching, inclusive, and iterative process, with three phases over a 6-year period (from funding in 2016 to launch in 2021). The development phases with, and for, Aboriginal and Torres Strait Islander communities involved (1) initial focus groups (30‐45 min, n=15) conducted by an Aboriginal researcher across 6 locations including metropolitan cities, rural regional towns, and towns servicing remote Aboriginal communities across Australia; (2) content and web development in collaboration with First Nations digital agencies; and (3) beta-testing of the web-based toolkit via a 15‐25 minute online survey to evaluate the toolkit’s acceptability, relevance, and usability.

**Results:**

Phase 1 community consultations (n=166) highlighted the need for culturally appropriate information and resources about methamphetamine use harms, impacts, and support options. This guided phase 2, where resources were developed to address knowledge gaps (eg, drug effects) and practical needs of communities (eg, yarning about drugs), along with content-specific meaningful First Nations artwork, interactive features, and other resources. Phase 3 beta-testing results were positive, with Aboriginal and Torres Strait Islander end users (n=73) reporting that the toolkit was easy to navigate and provided appropriate and relevant information. The majority (67/73, 91.8%) of participants reported that it was clear what information the toolkit provided, and most (54/73, 73.9%) were “very likely” to recommend it to others. Areas for improvement were identified (eg, explanation of artwork development and improving the clarity of text) and addressed before launch. The web-based toolkit was officially launched on July 21, 2021 (reach as of June 2025: >2.74M page views, >1.44M unique website users, and >366K hard copy resources distributed to >1793 organizations across Australia).

**Conclusions:**

It is critical that First Nations health resources are community-led and iteratively co-developed with experts and health workers supporting communities. Conducted over 6 years and involving >240 Aboriginal and Torres Strait Islander community members, Elders, experts, and health workers, such a process was used to develop this culturally appropriate, relevant, and acceptable web-based toolkit about crystal methamphetamine for Aboriginal and Torres Strait Islander peoples. While focused on an area of high need within Australia, this process provides an effective and strengths-focused roadmap to achieve this in other areas of health and in other countries working with First Nations peoples.

## Introduction

Methamphetamine is a global health concern with rates of use increasing. The 2024 United Nations World Drug Report estimated that 30 million people worldwide use amphetamine (including methamphetamine) [1]. In Australia, harms associated with methamphetamine use are high because crystal methamphetamine, often the more pure and potent form of the drug, is the most common type of amphetamine used [2]. There are indicators that methamphetamine use is more prevalent among some Aboriginal and Torres Strait Islander communities than non-Indigenous Australians [3]. For example, in 2022‐2023, a total of 2.7% of Aboriginal and Torres Strait Islander people reported using methamphetamine and amphetamine in the previous 12 months. This is estimated to be 2.3 times higher than the use of methamphetamine and amphetamine in non-Indigenous people [2]. While population prevalence estimates are limited by small sample sizes, research suggests that Aboriginal and Torres Strait Islander people can experience a disproportionate burden of harm from methamphetamine use. This may be due to the concurrent psychosocial and health stressors driven by the historic, political, and structural disadvantages that shape Aboriginal and Torres Strait Islander peoples’ everyday lives [4,5]. These harms are further exacerbated by a lack of accessible, evidence-based, and culturally appropriate resources for Aboriginal and Torres Strait Islander people directly or indirectly affected by methamphetamine use [3,5,6]. Despite these ongoing historical and structural challenges, Aboriginal and Torres Strait Islander communities demonstrate ongoing strength and resilience, as evident in sustained advocacy for culturally grounded, community-led healing and systemic change in health policy and practice [7]. Health resources and interventions that draw on the existing strengths, knowledge, and experiences of Aboriginal and Torres Strait Islander peoples are critical in empowering communities affected by methamphetamine use and enacting meaningful and sustainable change in health outcomes. Web-based and digital technology enables health information, resources, and support to reach large groups of vulnerable and geographically diverse communities, such as Aboriginal and Torres Strait Islander peoples [6,8-10]. The National Aboriginal and Torres Strait Islander Health Plan [11] highlighted the importance of e-Health technology and online services to ensure Aboriginal and Torres Strait Islander peoples have affordable and reliable access to evidence-based health information. Recent national data indicate high levels of internet access among Aboriginal and Torres Strait Islander peoples. In 2022‐2023, a total of 88.3% of Aboriginal and Torres Strait Islander people aged 15 years and over reported using the internet in the previous three months, with 81.3% reporting daily internet use, although substantial disparities persist by remoteness and affordability [12,13]. This suggests that a web-based format is a viable avenue for disseminating health information on a large-scale. Further, engagement with social media for health resources and help-seeking has increased significantly among Aboriginal and Torres Strait Islander people in recent years [14-16]. Despite widespread agreement on the need for accessible, reliable health education, there is a lack of evidence-based, community-led, and co-developed resources available online regarding methamphetamine, particularly the crystal form. To address this resource gap, the Cracks in the Ice resources for Aboriginal and Torres Strait Islander peoples toolkit [17] was developed through a broad-reaching, inclusive, and iterative process, involving multiple phases over 6 years (2016‐2021).

The resources described in this paper are an extension of the Cracks in the Ice web-based toolkit [18], developed in 2015‐2016, and launched in 2017, to provide evidence-based, credible, and up-to-date information, resources, and support to Australian families and communities affected by crystal methamphetamine [19-21]. During the development of Cracks in the Ice, it was recognized that a separate community-led and driven, culturally appropriate resource regarding methamphetamine for Aboriginal and Torres Strait Islander peoples was needed. To meet this need, the Australian Government Department of Health provided additional funding to develop the community-led, culturally appropriate web-based toolkit, the first of its kind nationally and internationally.

Engaging in community-led processes and collaborating with Aboriginal and Torres Strait Islander communities in a genuine and meaningful way is crucial in developing health resources that are trustworthy, relevant, and reflect cultural values, beliefs, and traditions [9]. Previous research has detailed the importance of developing digital health resources and services with Indigenous communities, not only to ensure that the technology is acceptable and useful, but also to “decolonize” the development process [22-24]. This involves engaging with a range of stakeholders at all stages of development to enable and empower people to tailor resources to their needs. For example, this can include, but is not limited to, people with lived experience, family, health professionals, and Elders or original knowledge keepers (as appropriate). Research has shown that health resources and interventions that have Aboriginal and Torres Strait Islander leadership in this way are more likely to be holistic in nature, needs-based, and culturally appropriate, and therefore more readily trusted, adopted, and shared among communities who will benefit most [25-27].

This paper summarizes the three phases involved in developing the Cracks in the Ice resources for Aboriginal and Torres Strait Islander peoples toolkit: (1) community consultation, (2) co-development of resources, and (3) beta-testing. The overall aim of this project was to undertake community-led development of culturally appropriate resources. The specific aims were to (1) identify and understand the most important information and digital resource needs of Aboriginal and Torres Strait Islander peoples regarding crystal methamphetamine use and its harms (phase 1), (2) develop a wide range of resources in response to diverse community needs (phase 2), and (3) assess the acceptability, functionality, usability, and compatibility of the toolkit among community members via beta-testing before launch (phase 3).

## Methods

### Overview of the Co-Development Process

**Figure 1.**
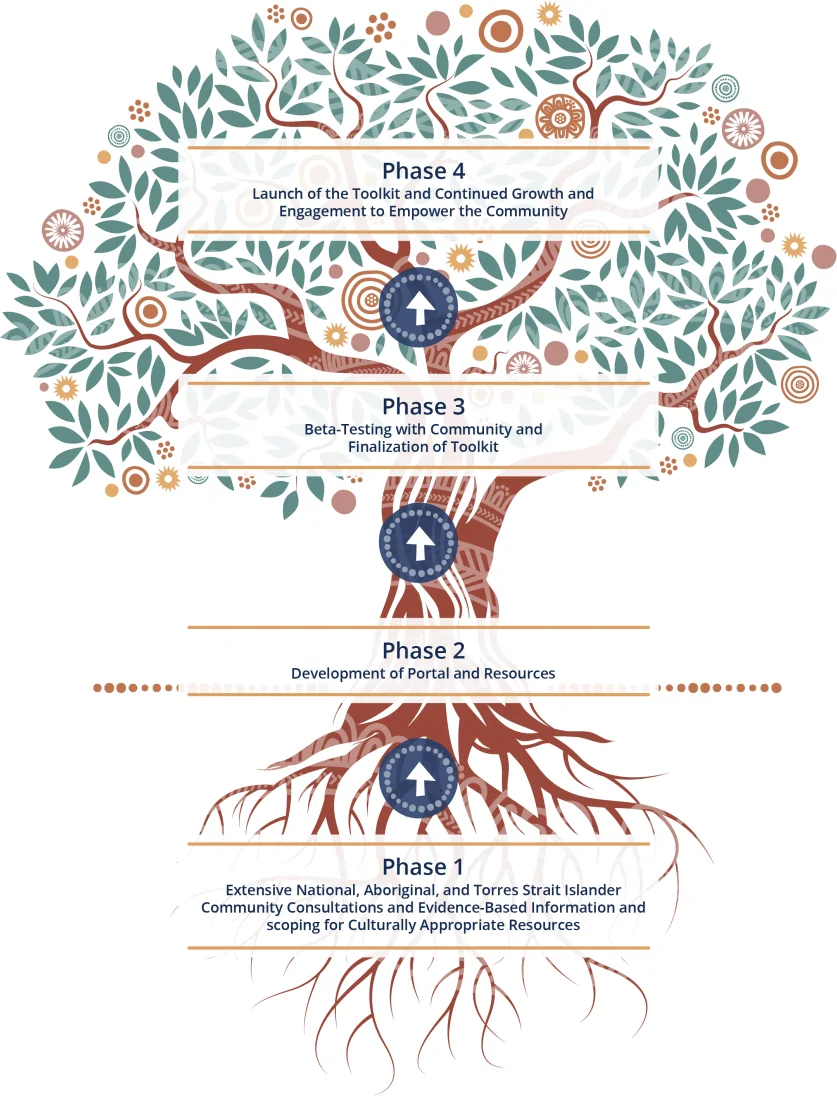
Overview of the development of the Cracks in the Ice resources for Aboriginal and Torres Strait Islander peoples toolkit.

Development of the Cracks in the Ice resources for Aboriginal and Torres Strait Islander peoples toolkit adopted a collaborative, community-led, and driven approach across all phases (Figure 1). The co-development process from project start to launch took place over 6 years (including disruptions to this project due to COVID-19 lockdowns and natural disasters). Similar to methods followed in the development of Cracks in the Ice [19], project directions, aims, and methodology were overseen by an expert advisory group (EAG) consisting of Aboriginal Elders, senior Indigenous and non-Indigenous researchers in the alcohol and other drug fields, as well as senior health professionals from a majority of Australian states and territories (except 1 state and 1 territory). EAG members were invited to join based on (1) experience in the area, (2) capacity to contribute, and (3) representing a broad cross-section of organizations serving metropolitan cities, regional towns, and rural and regional towns servicing remote communities across Australia. The EAG met regularly (annually until 2021, then biannually) with meetings chaired by an Elder, and they also provided ongoing input via email and surveys (eg, during phases 2 and 3).

The EAG provided, and continues to provide, guidance and advice throughout all phases of the toolkit development, maintenance, and dissemination. The EAG facilitated collaborations with people with lived experience of methamphetamine use to provide guidance throughout the development process. Partnerships were also established with Aboriginal Community Controlled Health Organizations (ACCHO’s) across Australia in a range of geographic areas.

**Figure 2.**
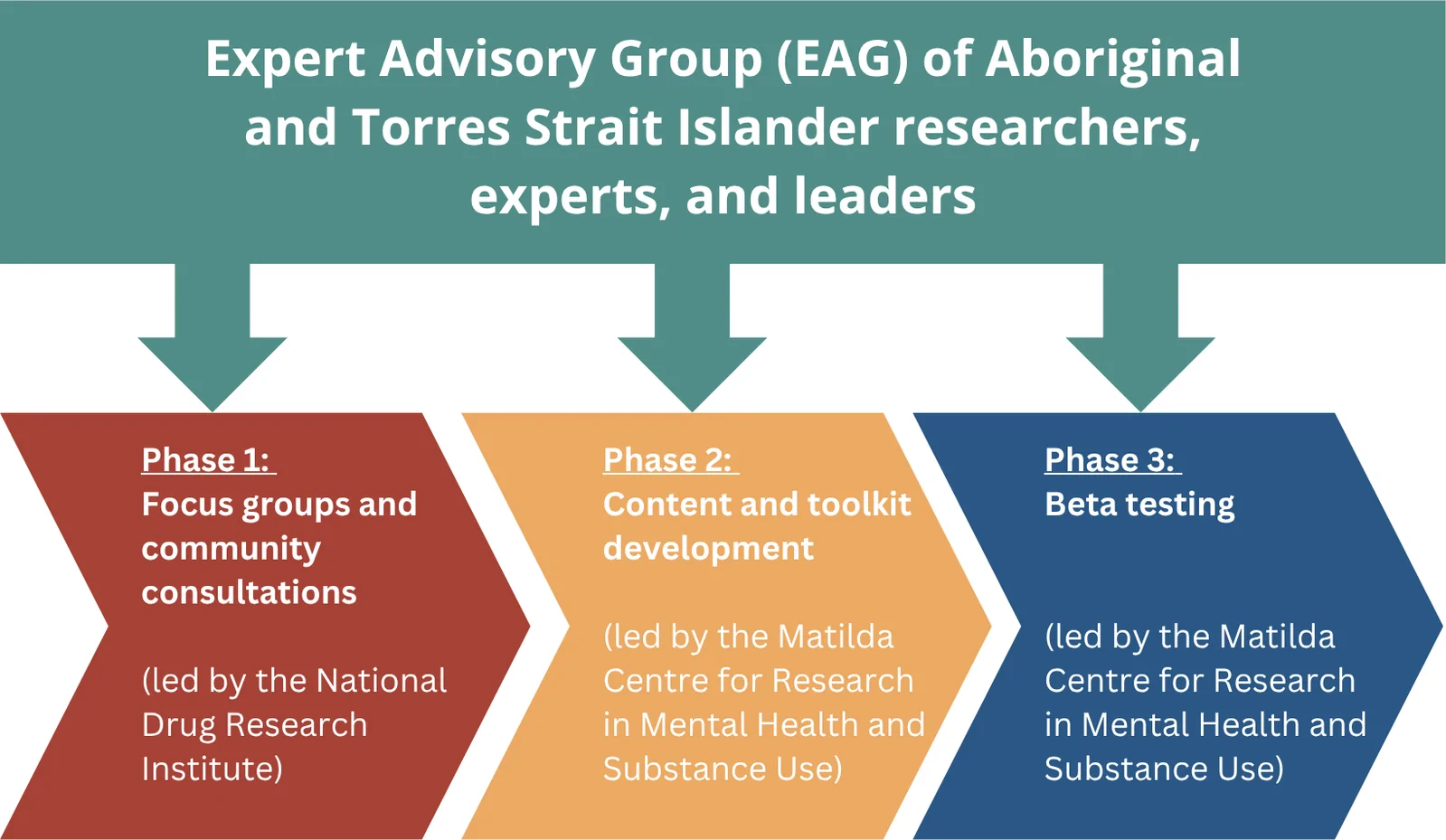
Co-development process of the Cracks in the Ice resources for Aboriginal and Torres Strait Islander peoples toolkit and study procedure.

Phase 1 was led by the National Drug Research Institute at Curtin University. Phases 2 and 3 were led by the Matilda Centre for Research in Mental Health and Substance Use at The University of Sydney, with the National Drug Research Institute team continuing in an advisory capacity (Figure 2).

### Phase 1: Initial End User Consultation

#### Ethical Considerations

Ethics approval was obtained through the Aboriginal Health Research Ethics Committee of South Australia (Protocol #04-17-724), Western Australian Aboriginal Health Ethics Committee (#820), Kimberley Aboriginal Health Planning Forum (2017‐15) in the case of Broome, Central Australian Human Research Ethics Committee (CA-17‐2875) and Cairns by the Far North Queensland Human Research Ethics Committee (HREC/17/QCH/73‐1156). Ethics approval was also obtained from Curtin University for each state-based HREC (HRE2017-0710, HRE2018-0029, HRE2018–0031, and HRE2017-0793). All participants provided informed consent.

#### Sample and Procedure

In 2018, focus groups (30‐45 min; n=15) were conducted in a variety of locations that covered metropolitan cities, rural regional towns, and towns servicing remote Aboriginal communities. These locations included Adelaide (Tarndanya; 1 group), Perth (Boorloo; 3 groups), Cairns (Gimuy; 5 groups), Albany (Kinjarling; 1 group), Alice Springs (Mparntwe; 2 groups), and Broome (Rubibi; 3 groups). Local ACCHO’s assisted in recruiting community members with insights from their own lived experience and that of their families, communities, or roles as health workers.

In each location, the number of focus groups and participants varied due to local factors at the time of the site visits.

The focus groups were facilitated by an Aboriginal researcher (Ms Sandra Taylor (ST)) to ensure cultural safety with the assistance of a non-Aboriginal researcher (KC). While funding was available to reimburse the substantial work of the Aboriginal facilitator, funding to reimburse focus group participants was not available. This was then explicitly incorporated into future grants. Thus, participation in the focus groups was voluntary, although transport assistance could be organized by focus group facilitators where needed.

The first three focus groups across two locations (Adelaide and Alice Springs) involved (1) participants identifying community needs with respect to crystal methamphetamine resources as well as (2) viewing the existing Cracks in the Ice web-based toolkit. After reviewing insights from these focus groups, the EAG highlighted that, in addition to adapting existing resources, it would be valuable to develop new resources that are not anchored to existing resources that were developed for non-Indigenous communities. Thus, the remaining 12 focus groups (Perth, Cairns, Albany, and Broome) adopted a variation of the World Café model and focused only on (1) identifying community needs with respect to crystal methamphetamine resources. The sessions incorporated appreciative inquiry, mind-mapping, and small group discussions and activities [28,29].

#### Data Analysis

Focus group sessions were audio recorded and transcribed verbatim by members of the research team (KC and ST). Transcripts were then deidentified and audio recordings deleted to safeguard participant information. Participant responses to focus group questions were also recorded on paper. Qualitative responses to (1) identifying community needs with respect to crystal methamphetamine resources were analyzed using thematic analysis [30] by an independent researcher (DG) not involved in the focus groups. Content relating specifically to the existing Cracks in the Ice resources obtained from the first three focus groups was not included. Data were analyzed using guiding principles from the 6-stage thematic analysis framework by Braun and Clarke [31] (1) reviewing data, (2) generating initial codes, (3) searching for themes, (4) reviewing themes, (5) defining and naming themes, and (6) writing the summary [32]. Raw data were reviewed line by line and coded into 72 categories based on similarity of words, phrases, and content (stages 1 and 2). These categories were then conceptualized into 10 broad groupings to identify major themes (stage 3). Themes were then independently reviewed and validated by KC and ST with the recordings and transcripts (stage 4). The final themes were discussed among DG, KC, and ST and amended based on consensus (stages 5 and 6).

### Phase 2: Content and Web Development

The development of content for the Cracks in the Ice resources for Aboriginal and Torres Strait Islander peoples toolkit began with a literature review and scoping exercise to identify existing resources (web-based and hard copy) for Aboriginal and Torres Strait Islander peoples about crystal methamphetamine [3]. Overall, the review identified limited availability of culturally responsive and co-developed resources on methamphetamine use developed in partnership with Aboriginal and Torres Strait Islander communities in Australia. Resources were developed or adapted to meet the community needs identified in phase 1, as well as to fill existing gaps identified by the scoping review. Prioritization of new content that addressed the needs of Aboriginal and Torres Strait Islander peoples, or content gaps identified by community members, was given (eg, social and emotional well-being, and tips for working with Aboriginal and Torres Strait Islander peoples). Resource development was conducted by the research team in collaboration with several Aboriginal-owned and led organizations specializing in creative design, website development, and language translations (eg, IB Creative, Iwiri, and Garuwa). The development process included adaptation of key fact pages (eg, what are the effects of ice?) to be culturally specific and appropriate, along with the development of new resources addressing community concerns including fact pages (eg, social and emotional well-being, and stages of change), animated videos, and other resources (including an interactive online community poster builder, as well as online and hard copy brochures). To assist with wider dissemination and cultural accessibility of resources, key content was translated into 3 Aboriginal and Torres Strait Islander languages: Warlpiri, Torres Strait Islander Creole by IB Creative, and Pitjantjatjara by Iwiri. These languages were chosen in consultation with the EAG as these three languages were among the most commonly spoken Aboriginal and Torres Strait Islander languages [33] and covered three different geographical areas of Australia.

### Phase 3: Beta Testing of the Web-Based Toolkit

#### Ethical Considerations

Ethics approval was obtained through the Aboriginal Health Research Ethics Committee of South Australia (Protocol # 04-20-879), Western Australian Aboriginal Health Ethics Committee (#1008), the Aboriginal Health and Medical Research Council of New South Wales (Protocol #1718/20), and the University of Sydney Human Research Ethics Committee (2020/693). All participants provided informed consent. Participants who completed the online survey were given the option to enter a draw to win one of twenty AUD $50 (a currency exchange rate of AUD $1=US $0.69 was applicable) groceries-only gift cards per state or territory (total of 60 gift cards nationwide).

#### Design and Procedure

Before launch, a beta version of the toolkit was tested with end users (November 2020 to March 2021) to ensure the toolkit aligned with the needs identified by the community in phase 1. Due to time constraints and ethical approvals, beta-testing in every state and territory was not feasible. Thus, South Australia, Western Australia, and New South Wales were selected in consultation with the EAG to incorporate feedback across metropolitan, regional, and remote areas.

This study was advertised through the Cracks in the Ice Facebook (Meta), X, formerly known as Twitter, pages, and e-newsletter, as well as through paid Facebook advertising to the general community but targeted to Aboriginal and Torres Strait Islander peoples (see Multimedia Appendix 1 for an example advertisement). This study was also shared with networks by the EAG, networks, and emails to Aboriginal Medical Services in respective states.

Participants were invited to complete an anonymous online REDCap (Vanderbilt University) survey [34,35] and provide feedback on an embedded beta version of the toolkit along with different information and design options. The survey was open to Aboriginal and Torres Strait Islander people residing in South Australia, Western Australia, and New South Wales, aged 18 years or older.

In the survey, participants responded to questions asking if they identified as a person who has used or uses methamphetamine, are a family member or friend of someone who has used or uses methamphetamine, are a health worker, and/or are part of a community where methamphetamine use is a concern. If participants responded “yes” to these questions, they were directed to questions related to the respective toolkit content for that group; otherwise, they were directed to the end of the survey.

The EAG provided ongoing input throughout the beta-testing phase of the toolkit, at annual EAG meetings, through regular email correspondence, and also completed the surveys on both the beta-version and the “live” web-based toolkit (one month before launch).

#### Measures

Respondents completed demographic questions about their age (“How old are you?”), gender (“Which of the following best describes your current gender identity? Female, male, non-binary/gender fluid, different identity (please provide)”), Aboriginal and Torres Strait Islander background (“Are you of Aboriginal or Torres Strait Islander origin?,”), geographical location (“What is your postcode?” and “From the map above, select the number that is closest to where you live”), and computer literacy (“How would you rate your skill in using a computer? Beginner, intermediate, advanced,” and “How do you usually access the internet? PC/desktop computer, smart phone, laptop, tablet”).

Respondents were also asked to respond to closed (eg, “Is it easy to see what information you can get from this page? Yes, No, why not - free text”) and open-ended (eg, “Please write any comments about what can be improved on the page for community members. For example, how the page looks, the use of artwork, pictures, graphics and names of headings”) questions about the design and cultural artwork, accessibility, and usefulness of content, including fact pages and brochures, were also included. In addition, the target groups for the toolkit (eg, people who use crystal methamphetamine, families and friends, health workers, and community members) were asked to provide feedback on specific resources designed for their respective needs.

#### Data Analysis

Beta-testing data were collated and analyzed in IBM SPSS Statistics (version 21.0). Descriptive analyses were conducted to capture key demographic data and summarize the absolute and relative frequencies of quantitative data. Open-ended responses were not analyzed using a formal qualitative analytic approach. Instead, selected participant quotes are presented verbatim to illustrate and contextualize the quantitative findings.

Usage data was collated and analyzed in Google Analytics 4. Figures for total users and views of all content housed in Cracks in the Ice resources for Aboriginal and Torres Strait Islander peoples toolkit were located through filtering website usage data by page path and screen class containing the site link [17].

## Results

### Phase 1: Initial End User Consultation

#### Participants

In total, 15 focus groups were attended by 166 participants (91/166, 54.8% female), with 10 participants attending 2 groups each. Participants included members of the communities where the focus groups were held, people with lived experience, health workers who attended as community members, and community members who attended as representatives of health service organizations. A small number of non-Indigenous service providers who worked closely with Aboriginal and Torres Strait Islander people attended some of the groups.

#### Key Areas of Community Concern

While there was a wide range of diverse thoughts and ideas, four key themes emerged and were agreed upon as important by the majority of participants: (1) harms to those who use crystal methamphetamine, (2) impact of crystal methamphetamine on families and communities, (3) availability and accessibility of services, and (4) supply and consumption of crystal methamphetamine. Within these themes, common topics related to early intervention and prevention, the need for success stories and messages of hope, overcoming stigma and structural barriers to accessing help, and the need for culturally appropriate services.

### Reflections on What Resources Were Needed

#### Content

The focus groups indicated resources in the toolkit should be tailored to health workers, family and friends, people who use crystal methamphetamine, and community members, rather than generic information across all groups. Key information needs related to effects and signs of use, prevalence of use, stages of change, and impacts of use on families. There was also feedback that the toolkit should provide information about locally based support services and how to navigate the health system.

Participants also provided suggestions for resources to assist with community-based prevention and harm reduction strategies, including practical information on how to prevent or reduce the harms associated with crystal methamphetamine use, managing acute behavioral incidents, and information or stories that break down stigma associated with crystal methamphetamine use to highlight a narrative of hope.

#### Presentation

Focus groups emphasized the importance of a culturally appropriate visual or pictorial format with interactive aspects including artwork and imagery, links to practical and relevant external websites and local services, ease of navigation, videos (eg, personal stories of hope), and localization through Aboriginal language.

### Phase 2: Content and Web Development

Driven by the findings from phase 1, the beta version of the Cracks in the Ice resources for Aboriginal and Torres Strait Islander peoples toolkit was developed in partnership with Aboriginal artists and/or designers, Aboriginal-led design agencies, and overseen by the EAG. The key content included in the beta-version is summarized in Table 1 below. See Multimedia Appendix 2 for a screenshot of the toolkit homepage before beta-testing.

**Table 1.** Summary of key content developed for Cracks in the Ice resources for Aboriginal and Torres Strait Islander peoples toolkit beta-version in response to identified community needs, along with rationale and partnered organizations.

Community need identified	Content developed	Rationale	Partnership with creative agency (details)
Aboriginal artwork throughout toolkit	A suite of design elements developed by an Aboriginal artist, including a “story behind the artwork” video [36] detailing the design journey and the meaning behind the artwork available to watch on the homepage	To increase cultural connection, enhance written content, and provide a cohesive, culturally relevant, and appropriate theme across the toolkit	Gilimbaa (Jenna Lee, Larrakia, Wardaman, and Karajarri woman)
Audio-visual medium	Six animations covering topic areas including “what is ice,” “the person behind the substance,” “using ice with other drugs,” “how to start a yarn,” “co-occurring conditions,” and “stigma and shame”	To increase accessibility and engagement with content for a diverse range of literacy abilities	Gilimbaa
Facts about methamphetamine, prevalence, effects and harms, how to help and respond	Twenty-eight fact pages adapted or developed on relevant topics, with many key pages also translated into 3 Aboriginal and Torres Strait Islander languages (Warlpiri, Torres Strait Islander Creole, and Pitjantjatjara)	To increase accessibility and assist with the dissemination of resources to diverse communities and languages	IB Creative (Warlpiri and Torres Strait Islander Creole), Iwiri (Pitjantjatjara)
Hard-copy materials and resources	Four hard copy brochures, one for each of the end user target groups (people who use crystal methamphetamine, family and friends, community members, and health workers), to be downloaded or ordered and delivered for free anywhere in Australia	To overcome digital and technological barriers and unreliable access to internet services, particularly for remote and rural communities	Gilimbaa
Interactive and adaptable elements	A “build your own poster” feature that allows community groups or organizations to create their own printable poster and fact pages with key evidence-based messages about crystal methamphetamine and its effects; an interactive embedded quiz on key facts about methamphetamine, its effects, and the law, with feedback and explanations for each question	To increase engagement and enable communities, including educators and health workers, to tailor information and resources to meet the unique needs of their communities	IB Creative, Netfront
Visual elements and localized images	Aboriginal-designed visual elements, graphics, and images of Aboriginal and Torres Strait Islander peoples, land, and place to accompany and enhance the text content on the toolkit	To increase cultural connection and engagement, create visual interest, and reduce the text load of web pages	IB Creative, Netfront

### Phase 3: Beta Testing of the Web-Based Toolkit

#### Participants

In total, 116 participants provided consent and started the beta-testing survey. Of them, 88 participants completed the full survey with 52 (44.8%) of these participants identifying as Aboriginal and/or Torres Strait Islander. An additional 28 people partially completed the survey (21 identifying as Aboriginal or Torres Strait Islander). No differences were found between the partially completed data and the completed data. This paper presents only data of Aboriginal and/or Torres Strait Islander participants (n=73; mean age 47, SD 12 years; 50/73, 68% female) as they are the primary target audience. Partial completions by Aboriginal and/or Torres Strait Islander participants were also included to capture as much relevant data as possible. Survey participants included people who had used crystal methamphetamine (n=19), family members and friends of people who use crystal methamphetamine (n=44), health workers (n=7), and community members (n=2). See Table 2 for a breakdown of participants and their experience with methamphetamine.

Most participants were from inner regional areas (21/73, 29%), followed by major cities (17/73, 23%) and outer regional areas (15/73, 21%), and 15% (11/73) of participants indicated that they spoke an Aboriginal and/or Torres Strait Islander language. Further, 41% of participants learned about the survey via social media. Participants accessed the internet primarily via smartphone (44/73, 60%), with 33% (24/73) using a laptop or desktop computer. Most participants (48/73, 66%) rated themselves as being at an “intermediate” skill level in using a computer.

**Table 2.** Summary of Aboriginal and/or Torres Strait Islander participants and their reported experience with methamphetamine. Participants were able to select multiple responses.

	Yes, n (%)
Have you ever used crystal methamphetamine (ice)?	19 (26.4)
Do you have a family member or friend who you think may be using ice?	44 (61.1)
Are you a health worker?	7 (9.7)
Has your community been affected by ice or are you concerned about ice use in your community?	2 (2.8)
None of the above	1 (1.4)

#### Feedback

Overall feedback from the survey was positive, and 67 of 73 (92%) participants reported that it was clear what information was available on the Cracks in the Ice resources for Aboriginal and Torres Strait Islander peoples toolkit. Further, 42 of 73 (58%) participants reported they were more likely to use this toolkit because of the cultural artwork. Most participants (54/73, 74%) indicated they would be “very likely” to recommend the toolkit to others. Additionally, 45 of 73 (66%) participants rated the cultural artwork as appropriate (“a little bit” or “very much so”) on a 5-point Likert scale. In providing feedback about the fact pages, one participant said, ”Well made & written. I think they have the potential to help a lot of people” (female with lived experience of methamphetamine use).

Another participant said, “Love that theres information on not just the effects of ice, but how to cut down on use and what happens, as well as how to stay safe and practical help” (female, family member). Feedback on the brochures was positive, with all participants reporting they liked how the brochure looked and found the information easy to read and understand. One participant said about the brochures, “particularly in Aboriginal community, hard copy is good, something that is visual…” (male, health worker).

For the health worker section, the majority (6/7, 86%) of health workers thought the landing page would be helpful for a health worker looking for resources about crystal methamphetamine to assist them in their practice. The majority (4/7, 57%) of health workers indicated that the information was relevant to them in their day-to-day work “every day.”

There were several areas identified for improvement. For example, multiple participants (n=10) highlighted the importance of acknowledging the origin and story of the artwork and artist,

*As an Aboriginal person, I do get concerned that we are using artwork which is interpreted differently depending on where you come from and cultural group. I always think it is good to acknowledge where the artwork comes from as it means different things to different cultural groups and could reduce confusion.*

[Male, health worker]

**Figure 3.**
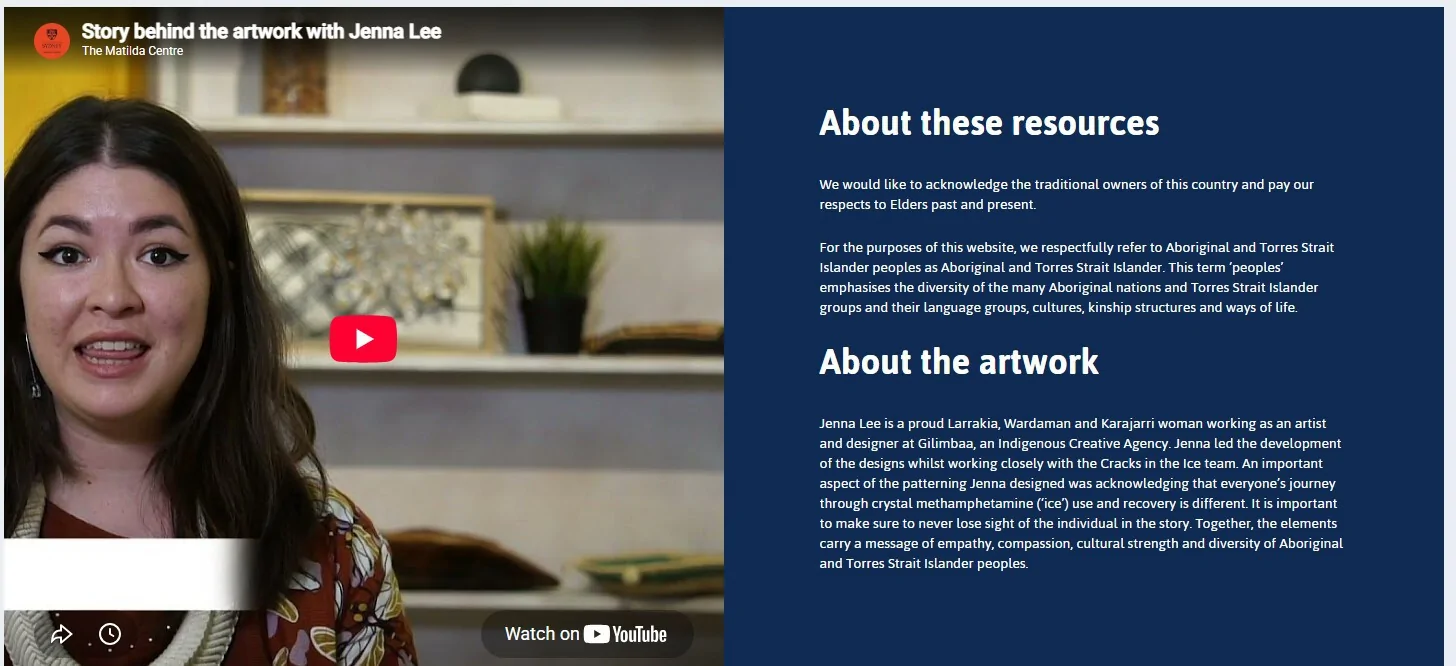
Screenshot of the bottom half of the Cracks in the Ice resources for Aboriginal and Torres Strait Islander peoples toolkit home page displaying video of the story behind the artwork.

In response to this feedback, a video of the artist detailing the story of the cultural elements was developed and placed prominently on the home page (Figure 3).

Another area for improvement identified by 6 participants, including people who use ice and health workers, was the need to increase accessibility of information through language and different formats,

*I think the fact sheets are appropriate in the length of time needed to read and digest the information. Some people may need support when going through the fact sheets so an audio option was able to be made that would also help those with low literacy and numeracy. Also if you are going to target remote communities it may be best to provide the audio in language where possible.*

[Male, health worker]

*It is easy to understand for someone who has already got some knowledge of how drugs work. For the target audience it may be difficult to understand or too long winded.*

[Female with lived experience of methamphetamine use]

Summary boxes to accompany the longer fact pages in the health worker section were also included.

Additionally, stories from real people impacted by methamphetamine use were highlighted as valuable by several people who use ice and family and friends (n=4). For example, one participant suggested, “You could put some personal stories on the effect’s of drug’s on themselves and their families” (female with lived experience of methamphetamine use). Another participant suggested, “Need stories from our mob who successfully give up the ice” (female, family member). The research team has since developed (with permission) scripted videos of lived experience stories based on information collected during beta-testing using actors in collaboration with GARUWA, a First Nations-led creative agency, launched in February 2025 [37]. On the beta version of the toolkit, the EAG reported that the health workers page and the family and friends landing page were “very” relevant for their respective intended end users. Suggested improvements included using fewer stock photography pictures, more images of real people, and more use of the cultural artwork across the toolkit. After viewing the “live” toolkit, EAG members reported they thought it was likely that people interacting with the toolkit would find the information “very useful.” One suggested improvement was for more support services to be listed. In response, the toolkit features the national ACCHO members list to ensure all support services listed are the Aboriginal community-controlled. In very remote areas where these may not be easily accessible, alcohol- and other drug-specific services have also been listed.

#### Usage Statistics

The web-based toolkit was officially launched on July 21, 2021. As of May 2025, the toolkit has had >1.44 million unique website users, >2.74 million page views, >366,000 hard copy resources distributed to >1793 organizations across Australia, including a range of both government-run services and Aboriginal Community Controlled Health Services. See Multimedia Appendix 3 for screenshots of the toolkit landing pages as of May 2025. Considering the entire lifespan of the website, the average reach per month is 22K unique users. While there is no reliable Australian benchmark for website visitors or page views for online health information resources specifically, according to a 2025 HubSpot report [38], the median estimate of unique visitors per month across all industries internationally is 20K, with the majority of websites across all sectors (46%) receiving between 1001 and 15,000 monthly visitors. These figures suggest that our website reach is above average. The toolkit for Aboriginal and Torres Strait Islander peoples has received 3 awards to date for First Nations Community impact and engagement.

## Discussion

### Summary of Findings

This paper described the community-led co-development of Cracks in the Ice resources for Aboriginal and Torres Strait Islander peoples, a web-based toolkit designed to provide accessible, evidence-based, culturally appropriate information and resources about crystal methamphetamine for Aboriginal and Torres Strait Islander people and their communities. The co-development process took place over 6 years and engaged more than 240 Aboriginal and Torres Strait Islander community members, Elders, leaders, and researchers across all phases of development.

Initial community consultations (phase 1) highlighted the significant impact of crystal methamphetamine on Aboriginal and Torres Strait Islander families, communities, services, and individuals, and the need for accessible and culturally sensitive information around methamphetamine. These findings are consistent with previous research indicating the significant impacts of methamphetamine use on Aboriginal and Torres Strait Islander individuals and communities, compounded by historic, social, and structural disadvantages [3-5,39]. Focus group participants provided valuable insight into the information and resources that were lacking for communities around methamphetamine, and highlighted the complex layers of culture, localization, empathy, and interactivity required to develop a resource toolkit that is accessible, appropriate, and acceptable for Aboriginal and Torres Strait Islander peoples and their communities.

The key considerations for development identified from phase 1 were, first, information and content that is relevant to the community, second, localized visual elements including pictures, artwork, and videos, and third, accessibility and ease of understanding for Aboriginal and Torres Strait Islander peoples. In terms of content, community members highlighted the need for practical information (eg, what is methamphetamine and how to prevent and reduce harms associated with methamphetamine use) as well as resources drawing on lived experience to combat stigma (eg, empathic and hopeful lived experience stories from Aboriginal and Torres Strait Islander peoples). Community members emphasized the need for toolkit content, structure, and presentation to be culturally appropriate through increased visual elements, including local imagery, video and audio mediums, as well as interactive components such as quizzes and links to Aboriginal and Torres Strait Islander-owned and run services.

It is of note that while this project was funded to develop a national portal, it is imperative to acknowledge the limitations of a universal “national” resource. While content and resources were developed to focus on overall issues identified by communities, localization was incorporated where possible. For example, the “build your own poster” activity aims to embed interactivity and flexibility by allowing people to tailor content for their communities’ needs. Similarly, the interactive service map allows people to select their local area. Further, we partnered with Aboriginal and Torres Strait Islander artists and creative agencies to develop visual design elements and artwork to be relevant to and reflect different Aboriginal and Torres Strait Islander communities. Along with this, a variety of images of Aboriginal and Torres Strait Islander people and Country were also included. In consultation with the EAG and Aboriginal organizations specializing in language translation, 28 fact pages were adapted or developed into 3 Aboriginal languages (Warlpiri, Torres Strait Islander Creole, and Pitjantjatjara) to assist with resource dissemination. The web-based toolkit also features a feedback loop with opportunities for end users to contact the research team, submit feedback on the toolkit, and suggest content they would like to see included in the toolkit. This feedback, along with input from the EAG and new evidence and research, is used to guide ongoing development of the toolkit to better meet the needs and reflect the strengths of Aboriginal and Torres Strait Islander peoples affected by methamphetamine.

### Community Response and Future Directions

Overall, the beta-testing results were positive, with the Cracks in the Ice resources for Aboriginal and Torres Strait Islander peoples toolkit demonstrating acceptability among Aboriginal and Torres Strait Islander end users. The hard copy brochures were also positively received by community members as a valuable resource to overcome limitations such as low digital literacy and unreliable access to the internet in remote and rural communities. Feedback was used to refine the toolkit to further enhance its relevance and appeal. For example, based on feedback on the length and density of information in the health worker section, summary sections at the top of fact pages were added. In response to requests for context and the story behind the artwork, a video featuring the artist who designed the visual elements across the toolkit was developed and placed on the toolkit homepage to acknowledge the artist and the cultural significance of the art.

The toolkit continues to be expanded and improved, for example, through the distribution and evaluation of lived experience videos featuring stories from Aboriginal and Torres Strait Islander people, which were co-developed with an Aboriginal-owned and led media agency. Launched in March 2025, these videos aim to address key consultation and beta-testing feedback on the importance and value of lived experience stories and voices from the community to dispel stigma and shame around methamphetamine use, instill hope for recovery and encourage help-seeking. A novel implementation study is in development to evaluate the efficacy of interacting with the Cracks in the Ice resources for Aboriginal and Torres Strait Islander peoples toolkit (including the Lived Experience videos) on knowledge and attitudes toward methamphetamine among Aboriginal and Torres Strait Islander peoples (Kershaw et al, unpublished data).

### Strengths and Limitations

This project should be considered in light of some limitations. First, the use of focus groups in phase 1 brings both advantages and caveats. In terms of limitations, as focus group discussions depend on participant dynamics, there is a risk of social-desirability bias, groupthink, and biasing certain dominant views over others [40]. This may be a particular issue around the discussion of sensitive and stigmatized topics such as illicit substance use within small or tight-knit communities. However, focus group discussions are also a valuable way to efficiently gather beliefs, attitudes, and insights into community needs from diverse participants, generating debate and encouraging participants to explore and clarify their perspectives [41,42]. The shift in methodology for 12 of 15 focus groups in phase 1 may have introduced bias into the results, and although the team did not observe any thematic shifts in data obtained from the first 3 to the last 12 groups, this was not formally assessed. Additionally, across all phases, budget constraints limited the scope and activities that could be undertaken. For example, in phase 1, reimbursement of participants was not possible, and this may have impacted recruitment and participation. Beyond this, we recognize that the absence of reimbursement may contribute to longstanding practices of lack of reciprocity in research with First Nations communities. While we sought to redress this in later phases of this project, including in the development and dissemination of the toolkit itself (eg, free distribution of hard copy resources and community webinars, and employment of an Indigenous Engagement officer to lead development and promotion), it does not represent the best practice in community research across all stages of this project. Second, the sample for phase 3 beta-testing was relatively small and recruited via convenience sampling, limiting generalizability, particularly considering the diversity of Aboriginal and Torres Strait Islander peoples across Australia. This further highlights the importance of ongoing input from Aboriginal and Torres Strait Islander peoples to enhance the cultural responsiveness and usefulness of the toolkit. Finally, it is important to acknowledge the cultural caveats of the research and co-development processes behind the toolkit. While the team adopted a culturally sensitive and Indigenous-led approach to all stages of developing the toolkit, which prioritized Aboriginal and Torres Strait Islander voices and perspectives, there are limitations and inherent biases that arise from the involvement of non-Indigenous researchers leading certain aspects of development. This includes the role of the (Indigenous and non-Indigenous) researchers influencing the final decision-making around the prioritization of toolkit content and features, impacting the extent to which community members were able to determine the final features of the toolkit. Further, interpretation and write-up of the data were led by a mix of Indigenous and non-Indigenous researchers, and the authors acknowledge the structural power imbalances, sociocultural privilege, and biases that may inadvertently influence this approach to the analysis of Aboriginal and Torres Strait Islander Peoples’ talk and text [43]. Finally, phase 3 beta testing has several limitations that should be considered when interpreting findings on toolkit acceptability. Although 116 participants commenced the survey, only 52 (44.8%) completers identified as Aboriginal and/or Torres Strait Islander, despite this being the primary intended audience. While the inclusion of partial responses increased the analytic sample to 73 and allowed broader inclusion, this choice may have introduced response bias, as incomplete surveys may reflect differing engagement or perceptions of relevance. Furthermore, the sample was heavily skewed toward family members and friends, with relatively limited participation from health workers and community members. Given that the toolkit contains sections specifically tailored to each of these groups, the small number of responses from health workers and community members constrains confidence in the acceptability and relevance of those components. As a result, findings from phase 3 primarily reflect the perspectives of family and friends and should not be assumed to generalize evenly across all intended user groups. Future evaluation should prioritize purposive recruitment of underrepresented groups, particularly health workers and community members, to strengthen conclusions regarding the toolkit’s acceptability and usability across its full target audience.

Despite these limitations, the Aboriginal and Torres Strait Islander leadership at both the academic and community level (including research team, staff, and focus group facilitators) was critical to the successful development of the toolkit and offers important lessons for future research with and for Indigenous communities both in Australia and internationally. The EAG and Aboriginal and Torres Strait Islander researchers and experts provided leadership and oversight across all stages of the development of the toolkit, from the conception of the toolkit to content development to the collection, analysis, and interpretation of beta-testing data. Adopting this community-led approach of co-development in health research and translation is critical to shifting the power and authority to Aboriginal and Torres Strait Islander communities and closing the gap in Aboriginal health [44]. This approach reflected the focus of the team (guided by the EAG) on decolonizing research and development practices, prioritizing and respecting Aboriginal and Torres Strait Islander voices and perspectives across all stages of research. Cultural safety and sensitivity were actively promoted through key safeguards, including employing Aboriginal facilitators and interviewers, hosting focus groups within ACCHOs, and ensuring participants were aware of culturally appropriate support services for any distress or concerns arising during participation. While this project reflects a step toward a more culturally responsive and representative research landscape, there remains significant progress to be made in relation to Aboriginal leadership in research. Time and budget constraints, short funding cycles, and staff turnover highlighted the pressing need for capacity building in Aboriginal professional and academic research roles and the ongoing challenges of integrating Indigenous knowledge, authority, and community expertise within the structural limitations of academic research. Finally, given the ongoing concerns around reliable access to services and information for Aboriginal and Torres Strait Islander peoples, particularly for those in remote and rural locations, a key strength of the Cracks in the Ice resources for Aboriginal and Torres Strait Islander peoples toolkit is accessibility, as it offers itself as a digital 24/7, free, and confidential source of information. Further, considering the significant barrier of stigma and shame that Aboriginal and Torres Strait Islander peoples continue to face around methamphetamine use, the option for private and confidential access to reliable information is crucial in overcoming barriers to health education and access to resources. The Cracks in the Ice resources for Aboriginal and Torres Strait Islander peoples toolkit addresses an area of high need, filling the gap in evidence-based, reliable information about methamphetamine developed by and for Aboriginal and Torres Strait Islander peoples and their communities.

### Conclusions

This study describes the community-led, in-depth, and iterative process of development and beta-testing of the Cracks in the Ice resources for Aboriginal and Torres Strait Islander peoples’ web-based toolkit. The toolkit provides culturally appropriate, evidence-based, and reliable information about crystal methamphetamine tailored to Aboriginal and Torres Strait Islander peoples, community members, family and friends, and health workers. Feedback from end users highlighted the important role of the culturally appropriate toolkit in meeting the needs of isolated and at-risk Aboriginal and Torres Strait Islander communities across Australia. The development of the Cracks in the Ice Resources for Aboriginal and Torres Strait Islander peoples demonstrates the strengths of community-led research and implementation, ensuring health information and education are co-developed with communities, for communities.

## Supplementary material

10.2196/75957 Multimedia Appendix 1 Recruitment advertisement.

10.2196/75957 Multimedia Appendix 2 Screenshot of Cracks in the Ice resources for Aboriginal and Torres Strait Islander peoples toolkit home page (at the time of beta testing).

10.2196/75957 Multimedia Appendix 3 Screenshots of Cracks in the Ice resources for Aboriginal and Torres Strait Islander peoples toolkit home page, family and friends landing page, health workers landing page, and community landing page (as of May 2025).

## Abbreviations

ACCHO: Aboriginal Community Controlled Health Organization
EAG: expert advisory group
