# Supplementary material for: Development of Cracks in the Ice Resources for Aboriginal and Torres Strait Islander Peoples, a Culturally Appropriate Evidence-Based Online Community Toolkit About Crystal Methamphetamine: Mixed Methods Study
Source: JMIR Form Res. 2026 Aug 21;10:e75957. doi: 10.2196/75957 (PMC13495995; doi:10.2196/75957)
Supplement: Supplementary file 1 [file formative-v10-e75957-s002.docx]

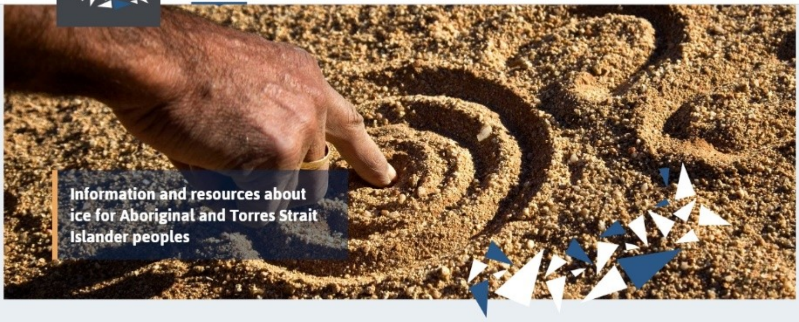

Information and resources about ice for Aboriginal and Torres Strait Islander peoples

FAMILY AND FRIENDS → Learn more

HEALTH WORKERS → Learn more

COMMUNITY MEMBERS → Learn more

Key fact

Methamphetamine comes in three different forms (ice, base and speed).

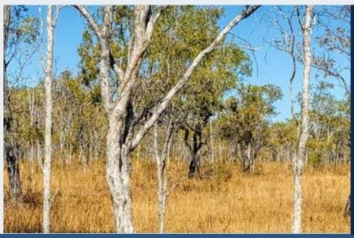

About these resources

Over the past 2 years we have been talking with Aboriginal and Torres Strait Islander peoples across Australia to understand what communities want to know about ice and how an online toolkit could support them in addressing ice related issues.

Based on the knowledge shared by community, we are developing a variety of resources guided by an expert advisory group of senior Aboriginal and Torres Strait Islander leaders.

We acknowledge the tradition of custodianship and law of the Country on which these resources and website are being built, the land of the Gadigal people of the Eora Nation. We pay our respects to their Elders, past, present and emerging.

We would like to acknowledge the traditional owners of this country and pay our respects to Elders past, present and emerging. Cracks in the Ice is committed to embracing diversity and eliminating all forms of discrimination in the provision of health services. Cracks in the Ice welcomes all people irrespective of ethnicity, lifestyle choice, faith, sexual orientation and gender identity.

© 2020 Cracks in the Ice. Copyright & disclaimer Privacy Design by Yump – Built by Netfront Follow us
