# Supplementary material for: Development of Cracks in the Ice Resources for Aboriginal and Torres Strait Islander Peoples, a Culturally Appropriate Evidence-Based Online Community Toolkit About Crystal Methamphetamine: Mixed Methods Study
Source: JMIR Form Res. 2026 Aug 21;10:e75957. doi: 10.2196/75957 (PMC13495995; doi:10.2196/75957)
Supplement: Supplementary file 1 [file formative-v10-e75957-s001.docx]

Twitter:

1/2 The new @cracksintheice toolkit has been developed to support Aboriginal & Torres Strait Islander peoples respond to crystal methamphetamine ‘ice’ use. Help improve the site by completing this survey! NSW survey: [Link to survey]. Links to SA and WA surveys below.

2/2 Complete this survey and go into the draw to win 1 of 20 grocery vouchers valued at $50.

SA survey: [Link to survey]

WA survey: [Link to survey]

Facebook:

The new @cracksintheice toolkit provides evidence-based information & culturally appropriate resources to support Aboriginal & Torres Strait Islander peoples respond to crystal methamphetamine ‘ice’ use. Would you like to contribute to the resource? Complete this survey and you could win one of 20 grocery vouchers valued at $50.

New South Wales: [Link to survey]

South Australia: [Link to survey]

Western Australia: [Link to survey]
