# Supplementary material for: Development of Cracks in the Ice Resources for Aboriginal and Torres Strait Islander Peoples, a Culturally Appropriate Evidence-Based Online Community Toolkit About Crystal Methamphetamine: Mixed Methods Study
Source: JMIR Form Res. 2026 Aug 21;10:e75957. doi: 10.2196/75957 (PMC13495995; doi:10.2196/75957)
Supplement: Supplementary file 3 [file formative-v10-e75957-s003.docx]

**Figure S1**. Screenshot of *Cracks in the Ice resources for Aboriginal and Torres Strait Islander peoples* toolkit home page (as of May 2025).

**Figure S2.** Screenshot of *Cracks in the Ice resources for Aboriginal and Torres Strait Islander peoples* family and friends toolkit landing page (as of May 2025).

**Figure S3.** Screenshot of *Cracks in the Ice resources for Aboriginal and Torres Strait Islander peoples* health workers toolkit landing page (as of May 2025).

**Figure S4.** Screenshot of *Cracks in the Ice resources for Aboriginal and Torres Strait Islander peoples* community toolkit landing page (as of May 2025).
